# Comparative metabolic profiling of *Cordia myxa* leaves and fruits based on UPLC-ESI/MS-MS analysis and investigation of antioxidant activities and enzyme inhibitory properties

**DOI:** 10.1038/s41598-025-04015-2

**Published:** 2025-06-02

**Authors:** Heba A. S. El-Nashar, Gokhan Zengin, Abdalrahman Tarek, Omayma A. Eldahshan

**Affiliations:** 1 Department of Pharmacognosy, Faculty of Pharmacy, Ain Shams University, Abbassia, Cairo, 11566 Egypt; 2 Department of Biology, Science Faculty, Selcuk University, Konya, Turkey; 3 Department of Pharmaceutical Chemistry, Faculty of Pharmacy, Ain Shams University, Abbassia, Cairo, 11566 Egypt; 4 Center for Drug Discovery Research and Development, Faculty of Pharmacy, Ain Shams University, Abbassia, Cairo, 11566 Egypt

**Keywords:** Antioxidant, *Cordia myxa*, Enzyme Inhibition, UPLC-ESI/MS-MS, Tyrosinase, Amylase, Cholinesterase, Plant sciences, Medical research

## Abstract

*Cordia myxa*, Assyrian plum, is an edible deciduous plant belonging to the family Boraginaceae and exhibited different traditional and pharmacological activities, including wound healing, emollient, expectorant, hepatoprotective, immune-modulator, and hypoglycemic. Our study aimed to investigate the metabolic profiling of *C. myxa* leaves and fruits growing in Egypt using the Ultra Performance Liquid Chromatography-Mass Spectrometry (UPLC-MS^n^) technique. Alongside, we determined its antioxidant properties and enzyme inhibitory activity against acetylcholinesterase (AChE), butyrylcholinesterase (BChE), tyrosinase, *α*-glucosidase, and *α*-amylase. UPLC-MS^n^ analysis revealed the identification of 24 and 19 compounds in the leaf and fruit extracts, respectively. The identified compounds cover different phytochemical classes like phenolic acids, flavonoids, phenolic glycosides, lignans, anthocyanins and fatty acids, flavonoids being the predominant one. The leaf extract showed stronger antioxidant properties (DPPH: 100.80 mg TE/g; ABTS: 134.43 mg TE/g, CUPRAC: 290.87 mg TE/g; FRAP: 205.78 mg TE/g), compared to the fruit extract (DPPH: 22.45 mg TE/g; ABTS: 91.74 mg TE/g; CUPRAC: 41.39 mgTE/g; FRAP: 33.63 mg TE/g). In contrast to other antioxidant assays, the metal-chelating ability of the fruit extract (20.59 mg EDTAE/g) was greater than that of the leaf extract (9.94 mg EDTAE/g). The AChE inhibitory effect of fruit extract (2.59 mg GALAE/g) was higher than that of leaf extract (2.51 mg GALAE/g), but the leaf extract (1.68 mg GALAE/g) showed a stronger BChE inhibitory effect than the fruit extract (1.25 mg GALAE/g). Further, the fruit extract showed almost 2.5 times greater tyrosinase inhibitory activity than the leaf extract. The *α*-amylase and *α*-glucosidase inhibitory activities of the leaf extract were superior to those of the fruit extract. The molecular docking studies showed high docking affinities between three compounds, quercetin, kaempferol, and caffeic acid, with all targeted enzymes. As a conclusion, *C. myxa* signifies an excellent natural supply of treasured bioactive constituents with promising antioxidant and enzyme inhibitory effects. Further phytochemical studies should be performed to isolate the responsible compounds and investigate their mechanism of action.

**Supplementary Information:**

The online version contains supplementary material available at 10.1038/s41598-025-04015-2.

## Introduction

Plants have been positioned as a primary treasure of bioactive compounds with promising medicinal and nutritional characteristics^1^. Historically, plant-derived materials were utilized as herbal supplements, botanicals, nutraceuticals, and drugs^2,3^. About 80% of the world’s population depends on natural sources for the management of several disorders^4,5^. Phytochemical research on plants is continuously performed to obtain promising naturally effective drugs for the treatment of different diseases^6,7^. It has been estimated that only 5–10% of the more than 250,000 higher plant species have been chemically explored^8,9^. By identifying phytoconstituents, they illustrate their approach to pharmacological knowledge and drug development, thus making medicinal plants a potential source of therapeutic agents for managing and preventing various disorders^10^. Plant-based antioxidants may explain the effectiveness of phytoconstituents, as they are believed to play a significant role in human health maintenance^11^. Antioxidant-rich medicinal plants are regarded as vital agents for mitigating diseases triggered by oxidative stress, such as diabetes, Alzheimer’s, Aging symptoms, hyperpigmentation, inflammatory diseases, hepatitis, and cancer^12^. One such genus of promising medicinal plants, *Cordia*, is a characteristic genus of flowering plants belonging to the family Boraginaceae^13^. Among the plants of the genus, there are four well-known species, including *C. myxa*, *C. dichotoma*, *C. latifolia*, and *C. Abyssinia*^14^. *C. myxa* grows up in the tropical and subtropical areas of America, Africa, Asia, and Oceania^15^. *C. myxa*, commonly named Assyrian plum, is an edible deciduous plant native to Asia^16^. It is well-known for its nutritional and medicinal value^17^. The fruits of *C. myxa* are used as vegetables, making pickles, and eaten fresh upon ripping^18,19^. In folk medicine, *C. myxa* has been utilized for different disorders such as wound healing, demulcent, anthelmintic, diuretic, astringent, emollient, expectorant, hepatoprotective, analgesic, immune-modulator, hypoglycaemic, anti-inflammatory, laxative, antioxidative stress, hypolipidemic, aphrodisiac, and antiulcer^20^. In the Unani System of medicine, the sticky and mucilaginous pulp of its fruit was recorded as Sapistan to be used in pharyngitis, coughs, sore throats, chest pain, and respiratory disorders^21,22^. Further, the fruits are rich in carbohydrates, phosphorus, and vitamin C (40 mg/100 g)^16^. From the phytochemical view, *C. myxa* was reported to be rich in numerous secondary metabolites such as phenolic acids, coumarins, tannins, resins, gums, mucilage, sterols, flavonoids, saponins, terpenoids, and oil^14^. Further, fruit is considered a rich source of nutrients like high dietary fibre, fructose, glucose, sucrose, minerals, fat, ash, carbohydrates, and proteins^23,24^. From the pharmacological value, the methanolic extract exhibited different biological properties such as antitumor, antioxidative, and anti-inflammatory^25^.

Oxidative stress could induce actual oxidizing and destructive sequences of reactions concerning the generation of reactive oxygen species (ROS) that exaggerate lipid peroxidation and thereby the progression of various chronic diseases^26^. Therefore, medicinal plants play a critical role in the treatment and prevention of a wide array of chronic diseases^27,28^. Diabetes mellitus is one of the destructive consequences of oxidative stress^29^. Furthermore, the phenolics-rich extracts were reported as cytoprotective agents that act by capturing the harmful oxidative stress, decreasing fat accumulation and hindering glucose and free fatty acid uptake by GLUT2 and CD36/FAT transporters^30,31^. Weakened insulin effectiveness resulted in high glucose concentrations, thereby worsening the oxidative stress status in the body^32^.

One of the recent applicable strategies for disease prevention or treatment is the inhibition of enzymes that control the pathogenesis of such disease^33^. For instance, inhibition of α-glucosidase and α-amylase, carbohydrate-metabolizing enzymes, can significantly decline the postprandial rise of blood glucose and therefore can be used as a therapeutic approach in the management of type 2 diabetic patients^34^. Formerly, several in vitro experiments have been accomplished proving potential α-amylase and α-glucosidase inhibitors from different food materials and edible plants such as cranberry^35^, *Cuscuta reflexa* (dodder)^36^, pepper^37^, soybean^38^, oregano^39^, and cranberry^35^. Similarly, the inhibition of enzymes like cholinesterase and tyrosinase could be a possible way for retardation of aging disorders including neurological diseases such as Alzheimer’s and skin diseases like hyperpigmentation^40,41^.

There is more than evidence that antioxidant and enzyme-inhibitory properties are greatly linked with the phytoconstituents of plant extracts. Hence, the main purpose of our study is to investigate the metabolic profiling of *C. myxa* leaves and fruits growing in Egypt via the Ultra Performance Liquid Chromatography (UPLC-MS^n^) technique. Alongside, we determined its antioxidant property and enzyme inhibitory activity against acetylcholinesterase (AChE), butyrylcholinesterase (BChE), tyrosinase,* α*-glucosidase, and *α*-amylase.

## Materials and methods

### Chemicals and reagents

The chemicals were purchased from Sigma-Aldrich (Darmstadt, Germany) including methanol (HPLC-grade), 2,2'-azino-bis(3-ethylbenzothiazoline-6-sulphonic acid (ABTS), 1,1-diphenyl-2-picrylhydrazyl (DPPH), gallic acid, rutin, electric eel acetylcholinesterase (AChE) (type-VI-S, EC 3.1.1.7), horse serum butyrylcholinesterase (BChE) (EC 3.1.1.8), galantamine, acetylthiocholine iodide (ATChI), butyrylthiocholine chloride (BTChI) 5,5-dithio-bis(2-nitrobenzoic) acid (DTNB), tyrosinase (EC1.14.18.1, mushroom), glucosidase (EC. 3.2.1.20, from *Saccharomyces cerevisiae*), amylase (EC. 3.2.1.1, from porcine pancreas), sodium molybdate, sodium nitrate, sodium carbonate, Folin-Ciocalteu reagent, hydrochloric acid, sodium hydroxide, trolox, ethylenediaminetetraacetate (EDTA), neocuproine, cupric chloride, ammonium acetate, ferric chloride, 2,4,6-tris(2-pyridyl)-s-triazine (TPTZ), ammonium molybdate, ferrozine, ferrous sulphate hexahydrate, kojic acid and acarbose. All chemicals were of analytical grade. Ethanol was purchased from El-Nasr Co., Cairo, Egypt.

### Plant material and extraction

The fresh leaves and fruits of *C. myxa* were obtained from El-Zohraya Garden, beside Cairo Tower, Giza, Egypt (0° 2′ 45.2976″ N and 31° 13′ 27.4476″ E) in February 2023. The leaves and fruits were kindly Mrs Therese Labib, Plant Taxonomy Consultant at the Ministry of Agriculture and El-Orman Botanical Garden, Giza, Egypt. A voucher specimen (Number: PHG-P-CM-522) is deposited at the Department of Pharmacognosy, Faculty of Pharmacy, Ain Shams University, Cairo, Egypt.

The plant leaves (1 kg) and fruits (500 mg) were chopped into small pieces and extracted separately with 70% ethanol (3 × 10 L). The pooled extracts were evaporated under reduced pressure at 55 °C using Buchi Rotavapor^®^ R-300 with Vacuum Pump V-700 (Buchi, Switzerland) until complete dryness to obtain 37 g and 10 g of brown materials for leaves and fruits, respectively.

### Determination of total phenolic and flavonoid contents

Total phenolics and flavonoids were quantified according to the procedures outlined by^42^. Gallic acid (GA) and rutin (R) were used as reference standards in the studies, with results expressed as gallic acid equivalents (GAE) and rutin equivalents (RE). The experimental details are given in the supplemental file.

### UPLC/MS^n^ analysis conditions

The phytochemical analysis of *Cordia myxa* extracts was assessed according to the previously reported method using high-performance liquid chromatographic (HPLC) analysis joined with an ESI-MS/MS spectrometer detector^43^. This technique allowed tentative identification of phytoconstituents based on the molecular weights. The plant extract (100 µg/ml) was dissolved in methanol (HPLC-grade) and then filtered via a membrane disc (0.20 μm). Then, the filtrate (10 µL) was injected into HPLC-ESI-MS/MS. The used HPLC instrument has the following specifications: Waters^®^ stocked with a reversed-phase C-18 column (ACQUITY UPLC-BEH C-18, particle size ~ 1.7 μm, dimensions = 2.1 × 50 mm). Before injection, the mobile phase was filtered through a membrane disc filter (0.2 μm) and sonicated. The elution period took 35 min using gradient elution (water and methanol acidified with 0.1% formic acid) with a flow rate of 0.2 mL/min. On an XEVO TQD triple quadrupole instrument, positive and negative ions were acquired using ESI-MS. Waters^®^ Corporation, Milford, MA 01757, U.S.A. supplied the HPLC unit and mass spectrometer. Edwards^®^, U.S.A., provided the vacuum pump at desolvation temperatures of 150 and 440 °C. The mass spectra were obtained using the software Masslynx 4.1 at an ESI range *m/z* of 100–1000. To tentatively identify the obtained mass spectra, the peak retention time (t_R_) and their fragmentation pattern were compared with the reported data in the literature.

### Determination of* in vitro* antioxidant capacity

In accordance with the methodologies detailed in our prior publication^44^, various antioxidant tests were carried out. The outcomes were represented as milligrams of Trolox equivalents (TE) per gram for the DPPH, ABTS radical scavenging, CUPRAC, and FRAP tests. In millimoles of TE per gram of extract, the phosphomolybdenum (PBD) test examined antioxidant potential, and in milligrams of disodium edetate equivalents (EDTAE) per gram of extract, the metal chelating activity (MCA) was determined. The experimental details are given in the supplemental file.

### Determination of inhibitory effects against some key enzymes

In accordance with the established protocols^44^, experiments on enzyme inhibition were performed on the samples. Acarbose equivalents (ACAE) per gram of extract were used to measure the activities that inhibit amylase and glucosidase, while milligrams of galanthamine equivalents (GALAE) per gram of extract were used to examine the inhibition of acetylcholinesterase (AChE) and butyrylcholinesterase (BChE). The amount of tyrosinase inhibition for each gram of extract was measured in milligrams of kojic acid equivalents (KAE). The experimental details are given in the supplemental materials.

### Molecular docking analysis

The docking studies were done using Discovery Studio 2.5.5, and Discovery Studio Visualizer 2016 was used to display the results. The 3D structures of the target proteins were downloaded from the Protein Data Bank (PDB) (http://www.rcsb.org). The target proteins are butyrylcholinesterase (PDB ID: 4BDS), acetylcholinesterase (PD ID: 4EY7), α-amylase (PDB ID: 5EMY), α-glucosidase (PDB ID: 5NN5), and tyrosinase (PDB ID: 6EI4). The downloaded target proteins were prepared by adding missing loops, protonating the proteins, standardizing amino acid names, insertion missing atoms in the amino acids, and applying the CHARMm forcefield. These were done using the prepare protein protocol. Tested compounds were docked in the same active pocket as the co-crystallized ligands. A prepare ligand protocol was used to prepare the compounds under investigation. Parameters were adjusted to remove any duplicates, fix bad valencies, generate tautomers, add hydrogen, and minimize energy. The CDOCKER algorithm was chosen for docking. Compounds were arranged according to their -CDOCKER energy. The higher the -CDOCKER energy, the better. Docking was validated by redocking of the co-crystallized ligands and calculating RMSD. RMSD numbers are 0.6294 for BChE (4BDS), 0.9583 for AChE (4EY7), 2.1403 for α-amylase (5EMY), 2.0257 for tyrosinase (6EI4), and 0.5326 for* α*-glucosidase (5NN5).

### Statistical analysis

All the experiments were carried out in triplicate, and the results are reported as mean ± SD. Statistical significance was assessed through the Student’s t-test (α = 0.05). Statistical analyses were carried out with GraphPad Prism (version 9.2).

## Results and discussion

### UPLC-MS^n^ tentative identification of plant metabolites of *C. myxa* leaves and fruits

**Fig. 1:**
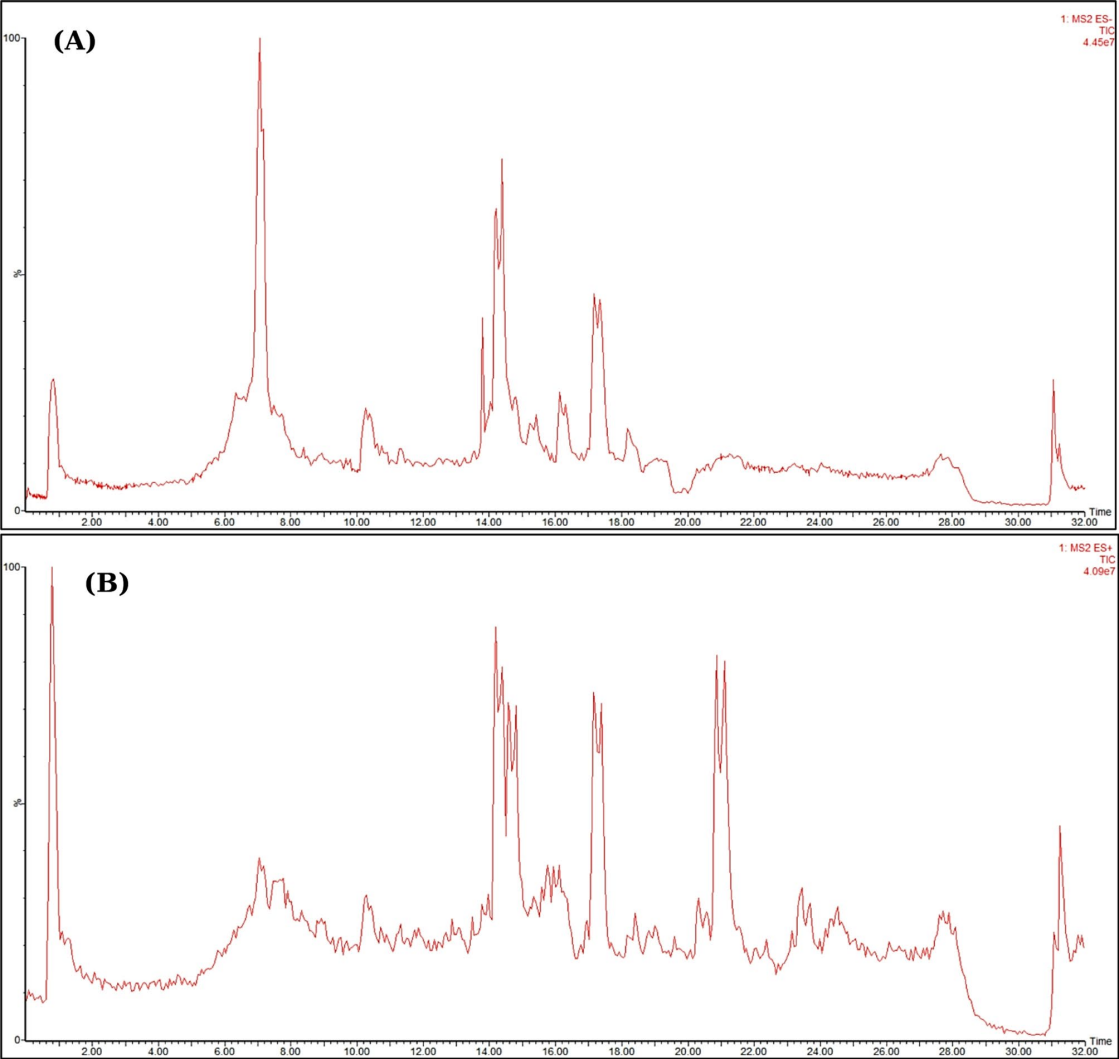
Total ion chromatogram (TIC) of leaf extract of *C. myxa* leaves (CML)in negative ion mode (**A**) and positive ion mode (**B**)

**Fig. 2:**
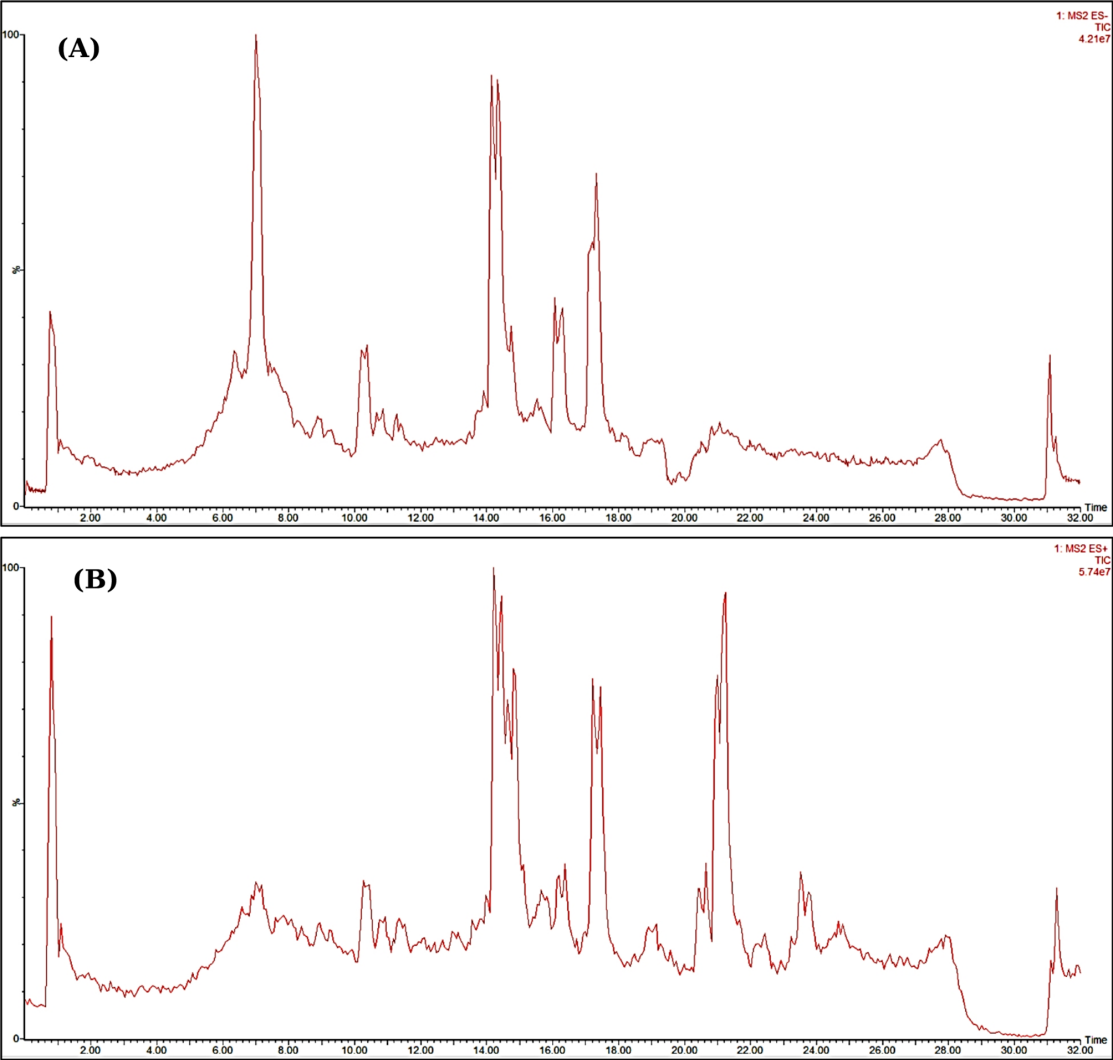
Total ion chromatogram (TIC) of fruit extract of *C. myxa* leaves (CML)in negative ion mode (**A**) and positive ion mode (**B**)

**Fig. 3:**
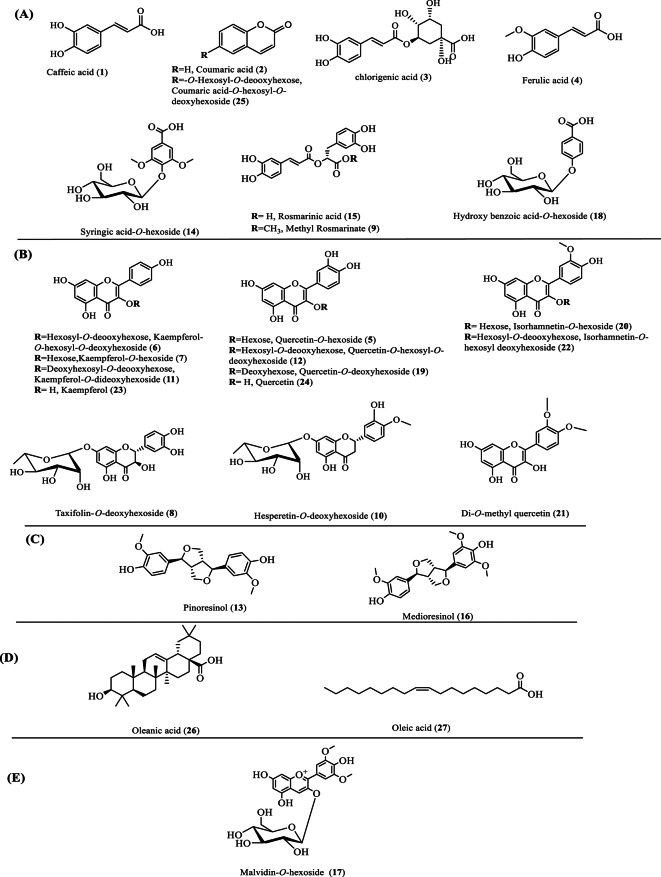
Chemical structures of metabolites identified in *C. myxa* leaves and fruits *using* UPLC-MS^n^: **A** phenolic acids, **B** flavonoids/flavonoid glycosides, **C** lignans, **D** fatty acids, **E** Anthocyanins

The phytoconstituents of the methanol extracts of *C. myxa* leaves (CML) and fruits (CMF) were tentatively characterized using ultra-performance liquid chromatography coupled with tandem mass spectrometry (UPLC/MS^n^) technique in the negative and positive ion modes (Figs. 1 and 2). After data manipulation, a total of twenty-four and nineteen chromatographic peaks were identified in the leaf and fruit extracts, respectively, as shown in Table 1. The metabolites were recognized based on the comparison of retention times, mass spectra data, and fragmentation patterns with previously reported data^45–47^. The identified metabolites, their molecular ions, fragment ions, molecular formula, and the underlying chemical class are shown in Table 1. The results showed that identified metabolites cover different phytochemical classes like phenolic acids, flavonoids, phenolic glycosides, lignans, anthocyanins and fatty acids. The compounds eluted in order of decreasing polarity, through which the phenolic acids were first to come, then flavonoid diglucosides, monoglucosides, aglycones, and fatty acids. The flavonoids were the predominant class of identified compounds in both extracts. Interestingly, this comparative phytochemical characterization is the first report on this species achieved *via* UPLC-MS analysis. The chemical structures of identified compounds in different classes are shown in Fig. 3. The detailed characterization of the plant metabolites is illustrated below according to their chemical class.

#### Flavonoids

Flavonoid glycosides are the most prevalent class in both extracts, denoted by thirteen chromatographic peaks (peaks **5**, **6**, **7**, **8**, **10**, **11**, **12**, **19**, **20** & **22**). Kaempferol, quercetin, and isorhamnetin displayed the main aglycone moieties in their glycosides. These glycosides were identified based on the loss of sugar moieties, and further characteristic Retro-Diels–Alder (RDA) fragmentation pattern of aglycone. The most intense fragment corresponding to the aglycone can be identified by removing the sugar moiety from *O*-glucosyl flavonoids^43,45,47^.

Kaempferol aglycone (*m/z* 286, C_15_H_10_O_7_) was shown in peaks **6**, **7**, and **11**, assigned as kaempferol-*O*-deoxyhexosyl-*O*-hexoside, kaempferol-*O*-hexoside, and Kaempferol-*O-*dideoxyhexoside, respectively. The fragmentation of the above-mentioned compounds resulted in the presence of kaempferol fragment [M-H]^−^ at *m/z* 285 due to cleavage of sugar molecules such as hexoses (glucose or galactose; 162 amu) and/or deoxyhexose (rhamnose; 146 amu)^45,47^. For example, peak **6** demonstrated a molecular ion peak in the negative ion mode at *m/z* 593(C_27_H_30_O_15_), with fragment ions at *m/z* 447 [M-deoxyhexose (146 amu)]^–^ and 285 [M-146-hexose (162 amu)]^–^, consistent with the predictable fragmentation outline of kaempferol-*O*-hexosyl-*O*-deoxyhexoside^48^. Similar fragmentation arrays have been noticed in peaks **12** and **22**, though with different aglycone parts, corresponding to quercetin and isorhamnetin, respectively.

Also, peak **5** (*m/z* 463, C_21_H_20_O_12_^–^), peak **7** (*m/z* 447, C_21_H_20_O_11_^–^), and **20** (*m/z* 477, C_21_H_20_O_12_^–^) was identified as quercetin-*O*-hexoside, kaempferol-*O*-hexoside and isorhamnetin-*O*-hexoside, respectively, due to cleavage of hexose moiety (162 amu), producing base ion peaks at *m/z* 301, 285, and 314, assignable to their aglycone parts. Similarly, as a result, of the removal of deoxyhexose sugar (146 amu), four compounds such as peaks **8** (*m/z* 449, C_21_H_22_O_11_^−^), **10** (*m/z* 448, C_22_H_24_O_10_^−^) and **19** (*m/z* 431, C_21_H_20_O_12_^−^) were recognized as taxifolin*-O*-deoxyhexoside, hesperetin-*O*-deoxyhexoside, and quercetin-*O*-deoxyhexoside, respectively.

Further, free aglycones were observed in peaks **23** (*m/z* 285, C_15_H_10_O_6_^−^) and **24** (*m/z* 301, C_15_H_10_O_7_^−^), corresponding to kaempferol and quercetin, respectively. Further, a methoxylated flavonol was identified at peak **21 (***m/z* 329, C_17_H_14_O_7_^−^), producing fragment ion peaks at *m/z* 314 and 301 due to sequential losses of two methoxy groups and interpreted as dimethoxy quercetin. With the same line of our findings, quercetin and kaempferol glycosides were previously isolated from different *Cordia* species, including *C. dichotoma*^20^ and *C. myxa*^49,50^.

#### Phenolic acids and phenolic acid derivatives

Phenolic acids and their derivatives demonstrated the second most predominant class after flavonoids. The UPLC-MS^n^ analysis revealed the characterization of five phenolic acids (peaks **1**, **2**, **3**, **4** & **15)**, assignable to caffeic acid, coumaric acid, chlorogenic acid, ferulic acid, and rosmarinic acid, respectively. Commonly, phenolic acids and their derivatives are tentatively described through the removal of water molecules (18 amu) and carboxylate part (44 amu) in their fragmentation spectra^1,51,52^. Accordingly, peak **1** (*m/z* 179, C_9_H_8_O_4_^−^) was spotted as caffeic acid due to the presence of fragment ion peaks at *m/z* 161 and 135, corresponding to the loss of water and carboxylate groups, respectively. Similar fragmentation profiles were observed in peaks **2**,** 3**, and **4**.

Also, a molecular ion peak (**15**) was found at *m/z* 359 in the negative ion mode and annotated as rosmarinic acid, which is an ester of caffeic acid and dihydroxy phenyl lactic acid)^53^. Accordingly, the presence of fragment ion peaks at *m/z* 197 (corresponding to dihydroxy phenyl lactic acid moiety) and *m/z* 179 (corresponding to caffeic acid moiety) confirmed the identification of this peak as previously reported in the leaves and fruits of *C. dichotoma*^20,14,25,54^. Further, peak **9** (*m/z* 373, C_19_H_18_O_8_^_^) was annotated as methyl rosmarinate due to the presence of fragment ion peaks at *m/z* 359 [M-CH_3_O^−^]^−^ and characteristic fragmentation pattern of rosmarinic acid (**15**) at *m/z* 197 and 179. As noted, methyl rosmarinate (**9**) was eluted early due to low polarity, compared to rosmarinic acid (**15**)^20^.

Moreover, three phenolic glycosides (peak **14**,** 18** & **25)** were identified as syringic acid-*O*-hexoside, hydroxybenzoic acid-*O*-hexoside, and coumaric acid-*O*-hexosyl-*O*-deoxyhexoside, respectively, based on the loss of hexose moiety (162 amu) and/or deoxyhexose (146 amu). Interestingly, these phenolic acids and their derivatives were previously reported in fruit and seed extracts of *C. myxa*^49,50^ and *C. dichotoma*^20,14,25,55^.

#### Furofurano-lignans

Two chromatographic peaks (**13** & **16**) were identified as pinoresinol and medioresinol, respectively, in both extracts. Peak **13** (*m/z* 357, C_20_H_22_O_6_^−^) showed a fragment ion peak at *m*/*z* 342 due to the removal of one methoxy group. Also, it showed characteristic fragment ions at *m/z* 151 (C_8_H_7_O_3_^_^) and 136 (C_7_H_4_O_3_^_^), resulting from the splitting of the tetrahydrofuran ring of pinoresinol^56^. Similarly, peak **16** (*m/z* 387, C_21_H_24_O_7_^−^) showed fragment ion peaks at *m*/*z* 372 [M-CH_3_]^_^ and 313 [M-3CH_3_-OH]. Also, it showed characteristic fragment ions of the tetrahydrofuran ring at *m/z* 151 (C_8_H_7_O_3_^_^) and 136 (C_7_H_4_O_3_^_^). With the same line of our findings, these compounds have been isolated from the fruits of *C. dichotoma*^25^.

#### Fatty acids

Two chromatographic peaks (**26** & **27**) were identified in the leaf extracts as fatty acids, namely, oleanic acid and oleic acid, respectively. Peak **26** (*m/z* 455, C_30_H_48_O_3_^−^) showed fragment ion peaks at *m/z* 409, 391 and 282, assigned to the loss of carboxyl, hydroxyl, and methyl groups, corresponding to oleanic acid. Peak **27** (*m/z* 281, C_18_H_34_O_2_^−^) was characterized as oleic acid, showing characteristic fragment ions at *m/z* 264, 222,135, 108, 95, corresponding to the loss of hydroxyl, carboxyl, and methyl groups, respectively. Interestingly, these fatty acids were previously identified in the seed extract of *C. myxa *^49,50^.

#### Anthocyanins

A chromatographic peak **17** (*m/z* 494, C_23_H_25_O_12_^+^) was defined as malvidin-*O*-hexoside, which showed its fragment ion peak at *m/z* 332 in positive mode ascribed to loss of hexose moiety (162 amu) and corresponding to malvidin.

**Table 1:** UPLC-ESI/MS-MS based characterization of phytoconstituents of the total extract of *C. myxa* leaves (CML; A) and fruits (CMF; B) in negative and positive ionization modes

No.	Compound name	Rt (min)	Molecular formula	Molecular weight	m/z detected and adduct		MS2 fragments	CML	CMF	Chemical class	Ref.
					[M-H]^−^	[M + H]^+^					
1.	Caffeic acid	0.77	C_9_H_8_O_4_	180	179	–	161,135	+	+	Phenolic acid	^49,50^
2.	Coumaric acid	0.86	C_9_H_8_O_3_	164	163	–	119	−	+	Phenolic acid	^57^
3.	Chlorogenic acid	2.52	C_16_H_18_O_9_	354	353	–	191, 179	+	+	Phenolic acid	^49,50^
4.	Ferulic acid	2.68	C_10_H_10_O_4_	193	194	–	178, 149, 134	+	**+**	Phenolic acid	^25^
5.	Quercetin-*O*-hexoside	5.08	C_21_H_20_O_12_	464	463	465	427, 301	+	**−**	Flavonol glycoside	^20^
6.	Kaempferol-*O*-deoxyhexosyl-*O*-hexoside	5.94	C_27_H_30_O_15_	594	593	594	447, 285, 242	+	**+**	Flavonol glycoside	^20^
7.	kaempferol-*O*-hexoside	6.32	C_21_H_20_O_11_	448	447	449	327, 285	+	+	Flavonol glycoside	^49,50^
8.	Taxifolin*-O*-deoxyhexoside	4.86	C_21_H_22_O_11_	450	449	451	285, 177	+		Flavanonol glycoside	^49,50^
9.	Methyl rosmarinate	5.82	C_19_H_18_O_8_	374	373	–	197, 179, 161	+	**−**	Phenolic acid derivative	^20^
10.	Hesperetin-*O*-deoxyhexoside	6.29	C_22_H_24_O_10_	449	448	451	301, 163, 150	+	−	Flavanone glycoside	^49,50^
11.	Kaempferol*O*di deoxyhexoside (Kaempferitrin)	6.55	C_27_H_30_O_14_	578	577	579	431, 285	+	+	Flavonol glycoside	^58^
12.	Quercetin-*O*-hexosyl-*O*-deoxyhexoside (Rutin)	6.63	C_27_H_30_O_16_	610	609	611	461, 301	+	+	Flavonol glycoside	^14^
13.	Pinoresinol	6.95	C_20_H_22_O_6_	358	357	359	342, 151, 136	+	**+**	Lignan	^25^
14.	Syringic acid-*O*-hexoside	7.19	C_15_H_20_O_10_	360	359	361	197, 153, 95	+	**−**	Phenolic glycoside	^55^
15.	Rosmarinic acid	7.22	C_18_H_16_O_8_	360	359	–	197, 179, 161	+	+	Phenolic acid	^14,20,25^
16.	Medioresinol	7.57	C_21_H_24_O_7_	388	387	–	372, 313, 151, 136	+	+	Lignan	^59^
17.	Malvidin-*O*-hexoside	7.97	C_23_H_25_O_12_^+^	493	492	494	332	+	−	Anthocyanin	^60^
18.	Hydroxy benzoic acid-*O*-hexoside	8.04	C_13_H_16_O_8_	300	299	–	137, 93	−	+	Phenolic glycoside	
19.	Quercetin-*O*-deoxyhexoside	12.25	C_21_H_20_O_12_	432	431	–	301	+	+	Flavonol glycoside	^20^
20.	Isorhamnetin-*O*-hexoside	13.81	C_22_H_22_O_12_	478	477.06	–	315, 300, 255	+	+	Flavone glycoside	^20^
21.	Di-*O*-methyl quercetin	14.37	C_17_H_14_O_7_	330	329	331	300	+	−	Methoxy flavonol	
22.	Isorhamnetin-*O*-hexosyl-*O*-deoxyhexoside	15.31	C_28_H_32_O_16_	624	623	625	315, 300	+	**+**	Flavone glycoside	^20^
23.	Kaempferol	17.31	C_15_H_10_O_6_	286	285	287	255, 227, 211, 159	+	+	Flavonol	^14^
24.	Quercetin	17.35	C_15_H_10_O_7_	302	301	303	245, 229, 179, 151	+	+	Flavonol	^14^
25.	Coumaric acid-*O*-hexosyl-*O*-deoxyhexoside	18.53	C_21_H_10_O_4_	472	471	–	325,307,163,145	−	**+**	Phenolic glycoside	^61^
26.	Oleanic acid	21.20	C_30_H_48_O_3_	456	455	–	409, 391, 282	+	**−**	Fatty acid	^62^
27.	Oleic acid	21.70	C_18_H_34_O_2_	282	281	–	264, 222, 135, 95	+	**−**	Fatty acid	^49,50^

### Determination of total phenolic and flavonoid contents

Polyphenols are the most important group of plant secondary metabolites and have been extensively studied for their biological activities, from antimicrobial to anti-inflammatory. Most studies have shown that dietary intake of polyphenols is inversely related to the development of chronic and degenerative diseases^63,64^. In this sense, most researchers have determined the content of phenolics to understand the biological potential of a plant extract. Therefore, in the current study, the total phenolic content (TPC) and flavonoid content (TFC) in the tested extracts were determined by colorimetric methods. As shown in Table 2, the total phenolics and flavonoids content was higher in the leaf extract (71.73 mg GAE/g; 13.85 mg RE/g) than in the fruit extract (17.51 mg GAE/g; 0.48 mg RE/g). In the literature, several studies have demonstrated the different content of phenols in the members of the genus *Cordia*. For example, El-Massry et al.^14^ reported that the total phenolic level in the fruit extract of *C. myxa* was 98.84 µg GAE/mg extract, and in the same study, the total flavonoid level was 22.40 µg QE/mg, which was higher than the results of the current study. In another study by Al-Musawi et al.^65^, the values were found to be 113.71 mg GAE/g and 68.9 mg RE/g. The total phenolic content was also reported by Kendir et al.^66^ as 401.09 mg GAE/g dry weight of *C. myxa*. Marini et al.^67^ worked on three *Cordia* (*C. dentata*,* C. megalantha*, and *C. bicolor*) species and the total phenolic levels varied between 40.52 mg GAE/g and 93.10 mg GAE/g. The differences could be explained by geographical and climatic conditions. In addition, spectrophotometric tests for determining total bioactive compounds have recently had some drawbacks. For example, in the Folin-Ciocalteu assay, not only phenols but also peptides can be reacted with the Folin-Ciocalteu reagent. Therefore, the results obtained may not reflect the actual level^68^. Therefore, to determine the differences in the extracts for the total bioactive compounds, chromatographic methods such as UPLC/MS^n^ were applied.

### Determination of antioxidant properties

Antioxidants neutralize free radicals and thereby control the radical chain reaction. This fact is a basis for the treatment of various serious diseases, including cardiovascular disease, diabetes, cancer, and inflammatory diseases^69^. With this in mind, the term “antioxidant” is one of the most common terms in scientific literature. To determine the antioxidant properties of *Cordia* extracts, we used various methods including free radical scavenging, reducing power and chelation tests. The results are summarized in Table 2. DPPH and ABTS are the best-known tests for examining the radical quenching ability of extracts. DPPH is a stable radical and reduces via hydrogen transfer from antioxidants, therefore, the color change is measured colorimetrically. ABTS is a cationic radical and is not stable, so we need to use it 12–16 h after preparing^70^. As can be seen in Table 2, the leaf extract (DPPH: 100.80 mg TE/g; ABTS: 134.43 mg TE/g) showed stronger radical quenching ability compared to the fruit extract (DPPH: 22.45 mg TE/g; ABTS: 91.74 mg TE/g). Another important mechanism is that the reduction ability of antioxidant compounds is related to their ability to donate electrons. High electron-donating ability reflects high antioxidant properties. For this purpose, CUPRAC and FRAP assays were performed, which include the conversion of Cu^2+^ to Cu^+^ and Fe^3+^ to Fe^2+^. Like the DPPH and ABTS results, the leaf extract (CUPRAC: 290.87 mg TE/g; FRAP: 205.78 mg TE/g) showed a stronger effect than the fruit extract (CUPRAC: 41.39 mgTE/g; FRAP: 33.63 mg TE/g). The phosphomolybdenum assay also involves the reduction of Mo(VI) to Mo(V) by antioxidants in the acidic state. The reducing ability of the leaf extract was higher than that of the fruit extract in the phosphomolybdenum assay. In contrast to other antioxidant tests, the metal chelating ability of the fruit extract (20.59 mg EDTAE/g) was greater than that of the leaf extract (9.94 mg EDTAE/g). Except for metal chelation, the results obtained are consistent with the total phenolic content, and therefore, the ability of the leaf extract could be attributed to its phenolic content. In addition, as shown in Table 1, some compounds, including quercetin-*O*-hexoside and methyl rosmarinate, were only detected in the leaf extract and these compounds could also contribute to the antioxidant activity^71,72^. The contradictory results for metal chelation can be explained by nonphenolic chelators such as peptides or polysaccharides^73^. It is likely that the content of polysaccharides in the fruit extract may be higher than in the leaf extract, and they could contribute to this ability. Several *Cordia* species have been studied for antioxidant properties in the literature. For example, Al-Musawi et al.^65^ reported that the ethanol extract of *C. myxa* fruit exhibited an 86.45% inhibition on DPPH at a concentration of 10 mg/ml. Samari et al.^74^ reported a significant reducing ability of the leaf extract of *C. myxa* in the FRAP assay. Khandelwal et al.^75^ found significant DPPH radical scavenging ability of ethanol (49.7%) and water extract (67.87%) from the leaf of *C. myxa* at a concentration of 2.5 mg/ml. Marini et al.^67^ reported that CUPRAC and FRAP abilities of three *Cordia* species were 229.82-661.82 mg TE/g and 129.20-370.46 mg TE/g, respectively.

**Table 2:** Antioxidant effects of *C. myxa* leaves and fruits extracts^*^

Samples	TPC (mg GAE/g)	TFC (mg RE/g)	DPPH (mg TE/g)	ABTS (mg TE/g)	CUPRAC (mg TE/g)	FRAP (mg TE/g)	PBD (mmol TE/g)	MCA (mg EDTAE/g)
Leaves	71.73 ± 2.27^a^	13.85 ± 0.18^a^	100.80 ± 0.08^a^	134.43 ± 0.17^a^	290.87 ± 5.38^a^	205.78 ± 4.04^a^	2.48 ± 0.07^a^	9.94 ± 0.66^a^
Fruit	17.51 ± 0.20^b^	0.48 ± 0.03^b^	22.45 ± 1.00^b^	91.74 ± 1.26^b^	41.39 ± 2.29^b^	33.63 ± 2.52^b^	0.95 ± 0.04^b^	20.59 ± 1.44^b^

Different letters indicate significant differences among the tested samples (*p* < 0.05)

TE: Trolox equivalent, EDTAE: EDTA equivalent

*Values are reported as mean ± SD of three parallel experiments

### Determination of enzyme inhibitory properties

As the prevalence of some diseases increases, public solutions become more urgent. For example, it is estimated that the prevalence of diabetes will reach 783 million people in 2045^76^. Likewise, the number of Alzheimer’s patients in developed countries is increasing day by day^77^. Currently, enzyme inhibition is one of the most widespread remedies on pharmacy shelves. By inhibiting important enzymes, the symptoms of the above-mentioned disease can be alleviated. In the treatment of diabetes mellitus, amylase and glucosidase inhibitors can delay the increase in blood sugar levels in diabetics^78^. Likewise, inhibition of cholinesterase can delay the hydrolysis of acetylcholine, and this fact can improve the cognitive function of Alzheimer’s patients^79^. Given the importance of inhibiting key enzymes, several compounds have been chemically prepared in the pharmaceutical industry, but their use raises some concerns (toxicity, gastrointestinal disorders, etc.). Therefore, novel, effective, and safe inhibitors are of great interest among researchers. Therefore, we investigated the enzyme inhibitory effect of *Cordia* extracts against cholinesterase, tyrosinase, amylase, and glucosidase. As shown in Table 3, the AChE inhibitory effect of fruit extract (2.59 mg GALAE/g) was higher than that of leaf extract (2.51 mg GALAE/g), but the leaf extract (1.68 mg GALAE/g) showed a stronger BChE inhibitory effect than the fruit extract (1.25 mg GALAE/g). Tyrosinase inhibition is associated with the treatment of hyperpigmentation problems and the tested fruit extract (65.04 mg KAE/g) had almost 2.5 times greater tyrosinase inhibitory activity than the leaf extract (26.63 mg GALAE/g). However, the amylase and glucosidase inhibitory activity of the leaf extract was higher than that of the fruit extract. As can be seen in Table 1, some identified compounds can be attributed to the observed enzyme inhibitory effect. Examples of effective inhibitors are caffeic acid^80–82^, rosmarinic acid^83–85^, and quercetin^86,87^. In the literature, several researchers reported the enzyme inhibitory effects of some *Cordia* members. Malik et al.^88^ reported that *C. dichotoma* significantly reduced AChE levels in the brain homogenates. The AChE, BChE, amylase, and glucosidase inhibitory effects of three *Cordia* species were 1.65–2.12 mg GALAE/g, 1.10–3.63 mg GALAE/g, 3.17–3.46 mmol ACAE/g, and 11.89–32.16 mmol ACAE/g, reported by Marini et al.^67^. In another study by De La Cruz-Jiménez^89^, the extract of *C. dodecandra* possessed weak glucosidase inhibitory effect (IC_50_ > 500 µg/ml). Taken together, the presented results can make a significant contribution to the pharmaceutical potential of *C. myxa* in the development of health-promoting applications.

**Table 3:** Enzyme inhibitory effects of the *Cordia myxa* leaves and fruits extracts^*^

Samples	AChE (mg GALAE/g)	BChE (mg GALAE/g)	Tyrosinase (mg KAE/g)	α-Amylase (mmol ACAE/g)	α-Glucosidase (mmol ACAE/g)
Leaves	2.51 ± 0.01^b^	1.68 ± 0.09^a^	29.63 ± 2.81^b^	0.16 ± 0.02^a^	1.71 ± 0.17^a^
Fruit	2.59 ± 0.01^a^	1.25 ± 0.17^b^	65.04 ± 1.87^a^	0.12 ± 0.01^b^	0.76 ± 0.01^b^

Different letters indicate significant differences among the tested samples (*p* < 0.05)

GALAE: Galanthamine equivalent, KAE: Kojic acid equivalent, ACAE: acarbose equivalent

*Values are reported as mean ± SD of three parallel experiments

### *In silico* molecular docking analysis

#### Assessment of docking affinities with 4BDS BChE

**Fig. 4:**
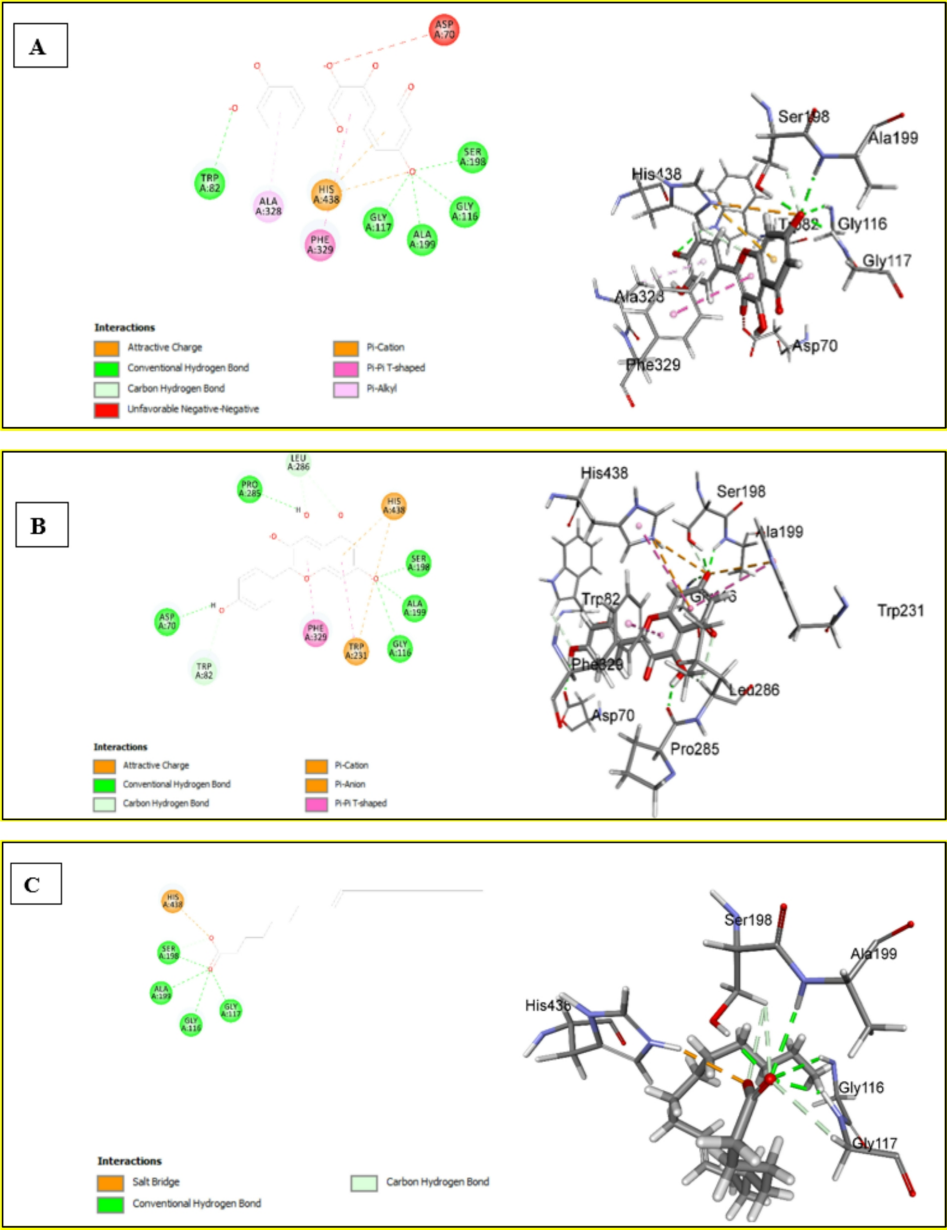
2D & 3D interaction of quercetin (**A**), kaempferol (**B**) and oleic acid (**C**) with 4BDS BChE

The CDOCKER algorithm was used to dock the tested compounds into the active pocket of 4BDS protein. The parameters were set to generate ten poses of each compound. The pose with the highest score was selected. This helps to have a glimpse of the binding mode and nature of binding of the investigated compounds. Table 4 shows the CDOCKER scores of the investigated compounds. The highest three compounds, quercetin, kaempferol, and oleic acid, have common interactions with common amino acids (Fig. 4A–C). The three compounds make conventional hydrogen bonds with GLY116, SER198, and ALA199. In addition, the three compounds participated in electrostatic interactions with HIS438. Quercetin and kaempferol make pi-cation and an attractive charge with HIS438, with quercetin making an additional carbon-hydrogen bond with HIS438, where oleic acid makes a salt bridge with the same amino acid HIS438. Besides the forementioned interactions, quercetin makes additional conventional hydrogen bonds with TRP82,* pi*-*pi* interaction with PHE329, and *pi*-alkyl interactions with ALA328. Kaempferol also makes pi-pi interaction PHE329 like quercetin. It also makes conventional hydrogen bonds with ASP70 and PRO285 and makes carbon carbon-hydrogen bond with TRP82 and LEU286.

**Table 4:** Docking scores of the major identified compounds with 4BDS BChE

No.	Compound	-CDOCKER Score
1	Quercetin	52.7248
2	Kaempferol	48.9772
3	Oleic acid	42.6859
4	Caffeic acid	34.1313
5	Isorhamnetin-*O*-hexoside	31.0807
6	Kaempferol-*O*-hexoside	27.3168
7	Oleanic acid	-80.0687
8	Isorhamnetin-*O*-hexosyl-*O*-deoxyhexoside	Failed to dock

#### Assessment of Docking affinities with 4EY7 ache

**Fig. 5:**
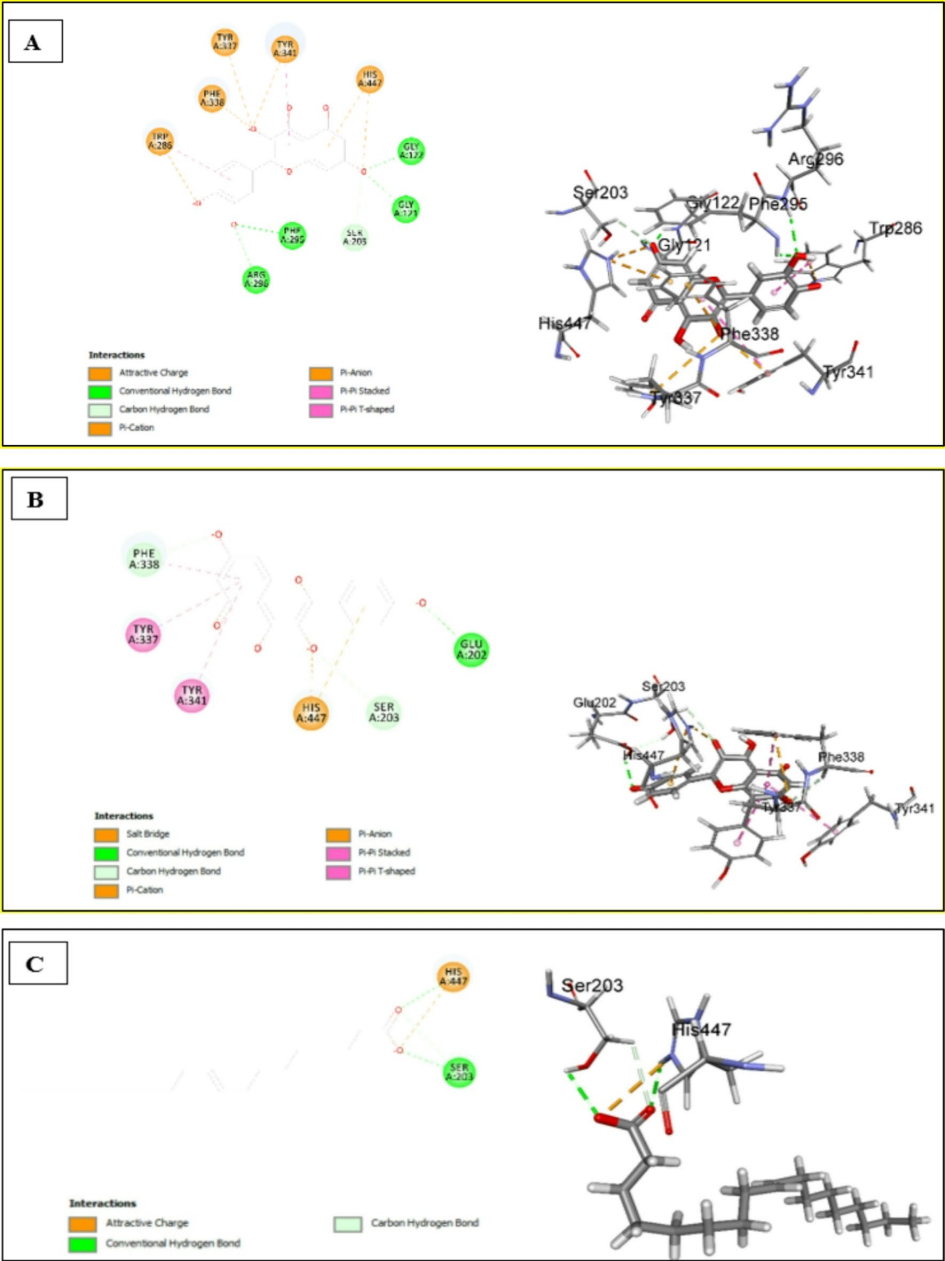
2D & 3D interactions of quercetin (**A**), kaempferol (**B**), and oleic acid (**C**) with 4EY7 protein

The investigated compounds were docked into the active pocket of 4EY7 AChE. Table 5 shows -CDOCKER scores of the compounds under investigation. As shown in Fig. 5A, conventional hydrogen bonds and electrostatic interactions are the main interactions of quercetin with the amino acids of the active pocket of 4EY7. It makes conventional hydrogen bonds with GLY121, GLY122, PHE295, and ARG296. It shows attractive charge and pi-anion interactions with TRP286, TYR337, PHE338, TYR341, and HIS447 in addition to pi-pi interactions with TRP286, PHE338, and TYR341. Kaempferol and oleic acid showed electrostatic interaction with HIS447 (Fig. 5B &C). Kaempferol also showed hydrogen bond interaction only with SER203, while oleic acid makes a conventional hydrogen bond with it. As illustrated in Table 5, isorhamnetin-*O*-hexosyl-*O*-deoxyhexoside shows weak affinity to the target (-CDOCKER score=-5.47526) while oleanic acid expresses the weakest affinity with -CDOCKER of -161.946. The interaction diagrams infer that HIS447 is a significant amino acid in the active site of the 4EY7 active pocket, where it is involved in interaction with every docked compound. Except for isorhamnetin-*O*-hexosyl-*O*-deoxyhexoside and oleanic acid, all the other compounds show good binding affinity and contribute to the activity against AChE.

**Table 5:** Docking scores of the major identified compounds with 4BDS ache

No.	Compound	-CDOCKER Score
1	Quercetin	45.6279
2	Kaempferol	43.3257
3	Oleic acid	39.199
4	Caffeic acid	32.5478
5	Kaempferol-*O*-hexoside	21.4887
6	Isorhamnetin-*O*-hexoside	15.4735
7	Isorhamnetin-*O*-hexosyl-*O*-deoxyhexoside	− 5.47526
8	Oleanic acid	− 161.946

#### Assessment of Docking affinities with 6EI4 tyrosinase

**Fig. 6:**
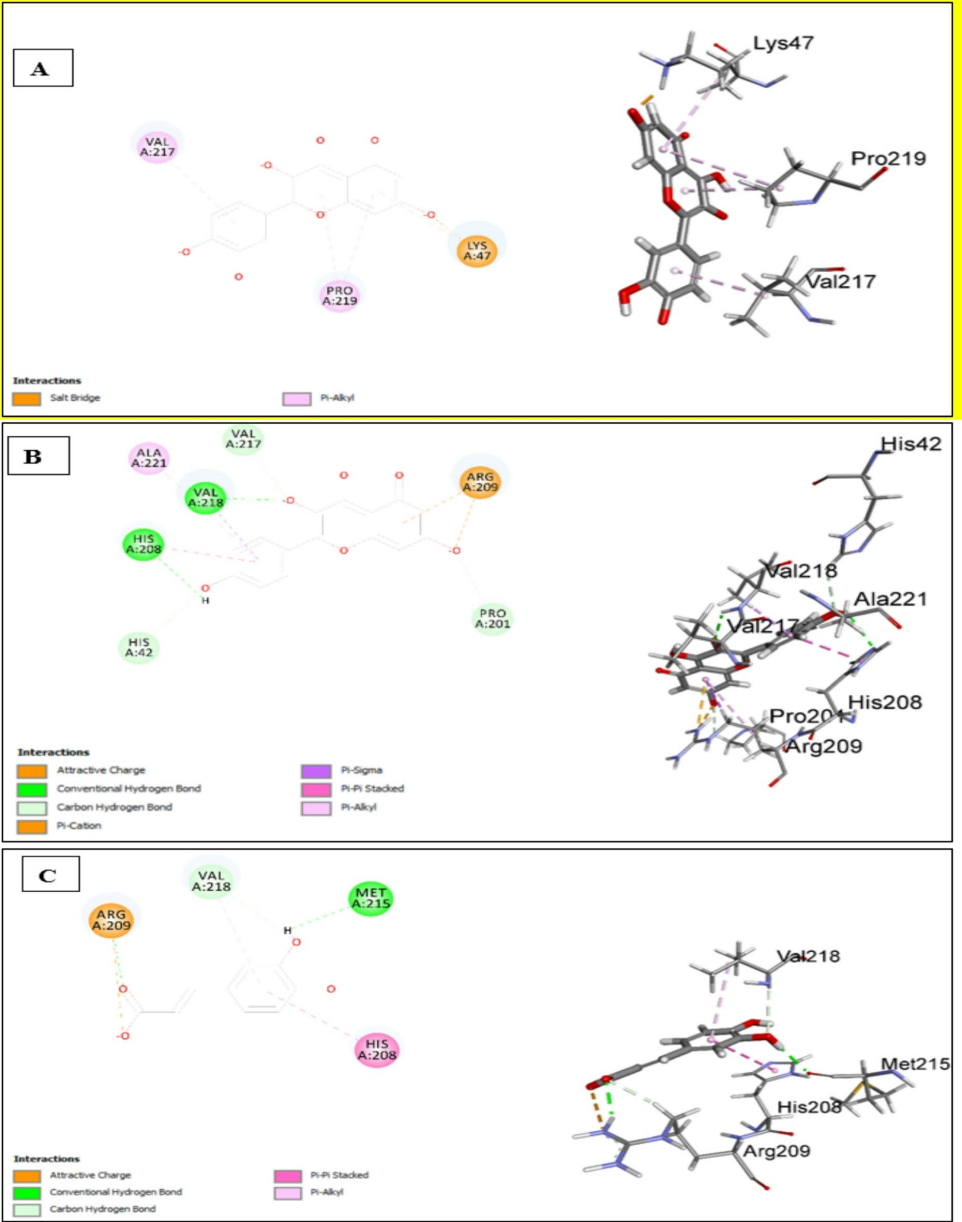
2D & 3D interactions of quercetin (**A**), kaempferol (**B**) and caffeic acid (**C**) with 6EI4 protein

As displayed in Table 6, quercetin showed the highest score of the docked compounds. The interaction diagrams (Fig. 6A) show two types of interaction salt bridge with LYS47, and a pi-alkyl interaction with VAL217 and PRO219. Kaempferol (Fig. 6B) makes conventional hydrogen bonds with HIS208 and VAL218, carbon-hydrogen bonds with HIS42, PRO201, and VAL217. It also shows attractive charge and pi-cation interaction with ARG209 in addition to *pi-pi* interaction, *pi*-alkyl interaction, and pi-sigma interaction with HIS208, ALA221, and VAL218. As illustrated in Fig. 6C, caffeic acid makes conventional hydrogen bonds with ARG209 and MET215, an attractive charge with ARG209, carbon carbon-hydrogen bond with VAL218, and a *pi*-*pi* interaction with HIS208. Oleanic acid expresses weak affinity, while Isorhamnetin-*O*-hexosyl-*O*-deoxyhexoside failed to dock. The scores illustrate that the first six compounds contribute to the activity against tyrosinase (Table 6).

**Table 6:** Docking scores of the major identified compounds with 6EI4 tyrosinase

No.	Compound	-CDOCKER Score
1	Quercetin	41.8888
2	Kaempferol	37.9299
3	Caffeic acid	30.8264
4	Oleic acid	30.7492
5	Kaempferol-*O*-hexoside	18.3843
6	Isorhamnetin-*O*-hexoside	9.69828
7	Oleanic acid	− 59.6035
8	Isorhamnetin-*O*-hexosyl-*O*-deoxyhexoside	Failed to dock

#### Assessment of docking affinities with 5EMY *α*-amylase

**Fig. 7:**
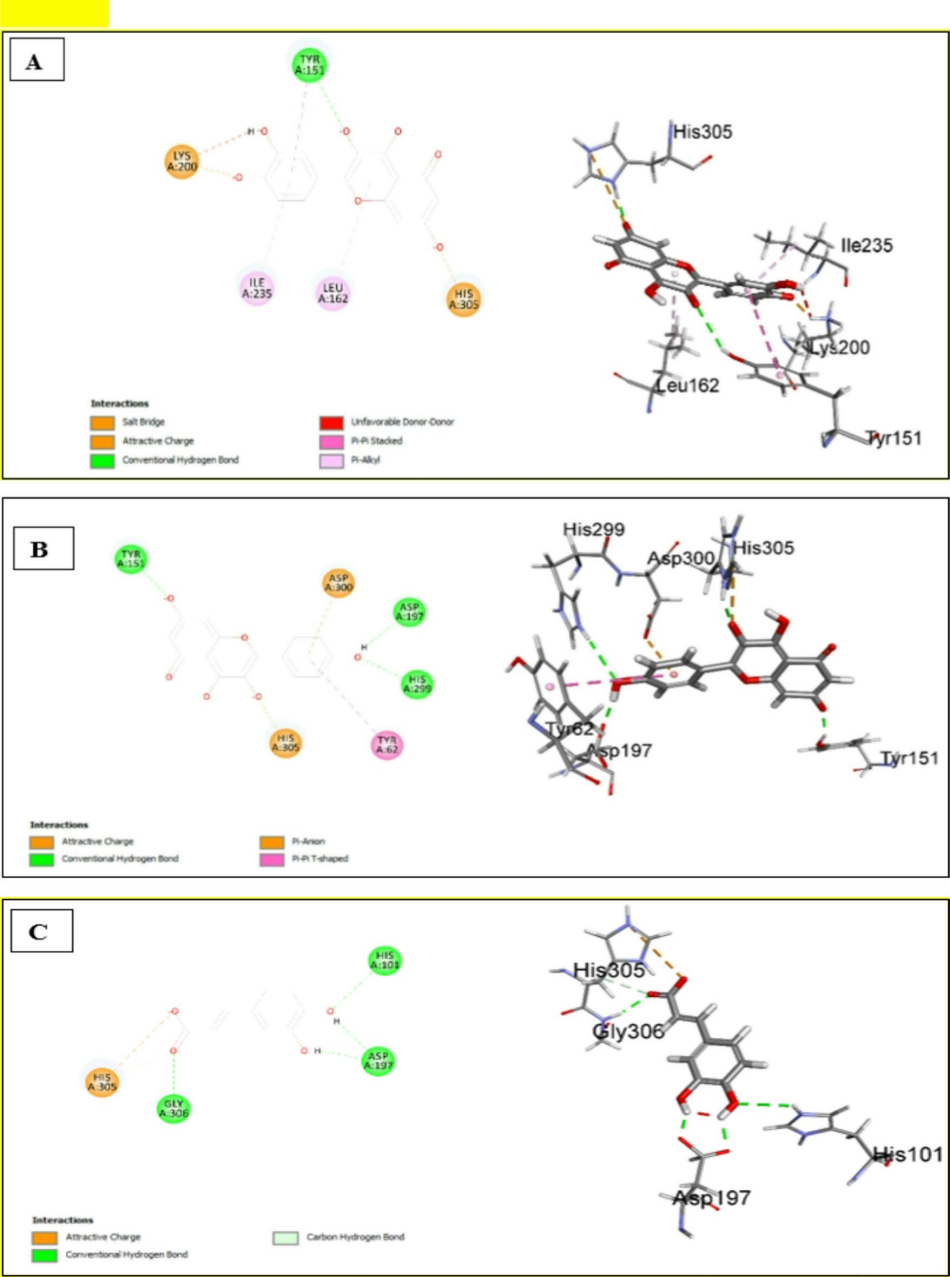
2D & 3D interactions of quercetin (**A**), kaempferol (**B**) and caffeic acid (**C**) with 5EMY α-amylase

The interaction diagrams of quercetin, kaempferol, and caffeic acid (Fig. 7) reveal that they have a common attractive charge interaction with HIS305. Quercetin and kaempferol also have a common conventional hydrogen bond with TYR151 (Fig. 7A, B). Also, both compounds displayed additional *pi-pi* interactions other than hydrogen bonds and attractive interactions. Caffeic acid exhibited a conventional hydrogen bond with GLY306 (Fig. 7C). Isorhamnetin-*O*-hexoside and kaempferol-*O*-hexoside have almost equal scores, 29.9239 and 29.8748, respectively (Table 7). Oleanic acid shows very weak affinity, whereas isorhamnetin-*O*-hexosyl-*O*-deoxyhexoside failed to dock. HIS305 seems to be a key amino acid in the active site as it is engaged in interactions with every docked ligand.

**Table 7:** Docking scores of the major identified compounds with 5EMY α-amylase

No.	Compound	-CDOCKER Score
1	Quercetin	53.8945
2	Kaempferol	45.1245
3	Caffeic acid	34.6329
4	Oleic acid	33.4752
5	Isorhamnetin-*O*-hexoside	29.9239
6	kaempferol-*O*-hexoside	29.8748
7	Oleanic acid	− 53.8138
8	Isorhamnetin-*O*-hexosyl-*O*-deoxyhexoside	Failed to dock

#### Assessment of Docking affinities with 5NN5 *α*-glucosidase

**Fig. 8:**
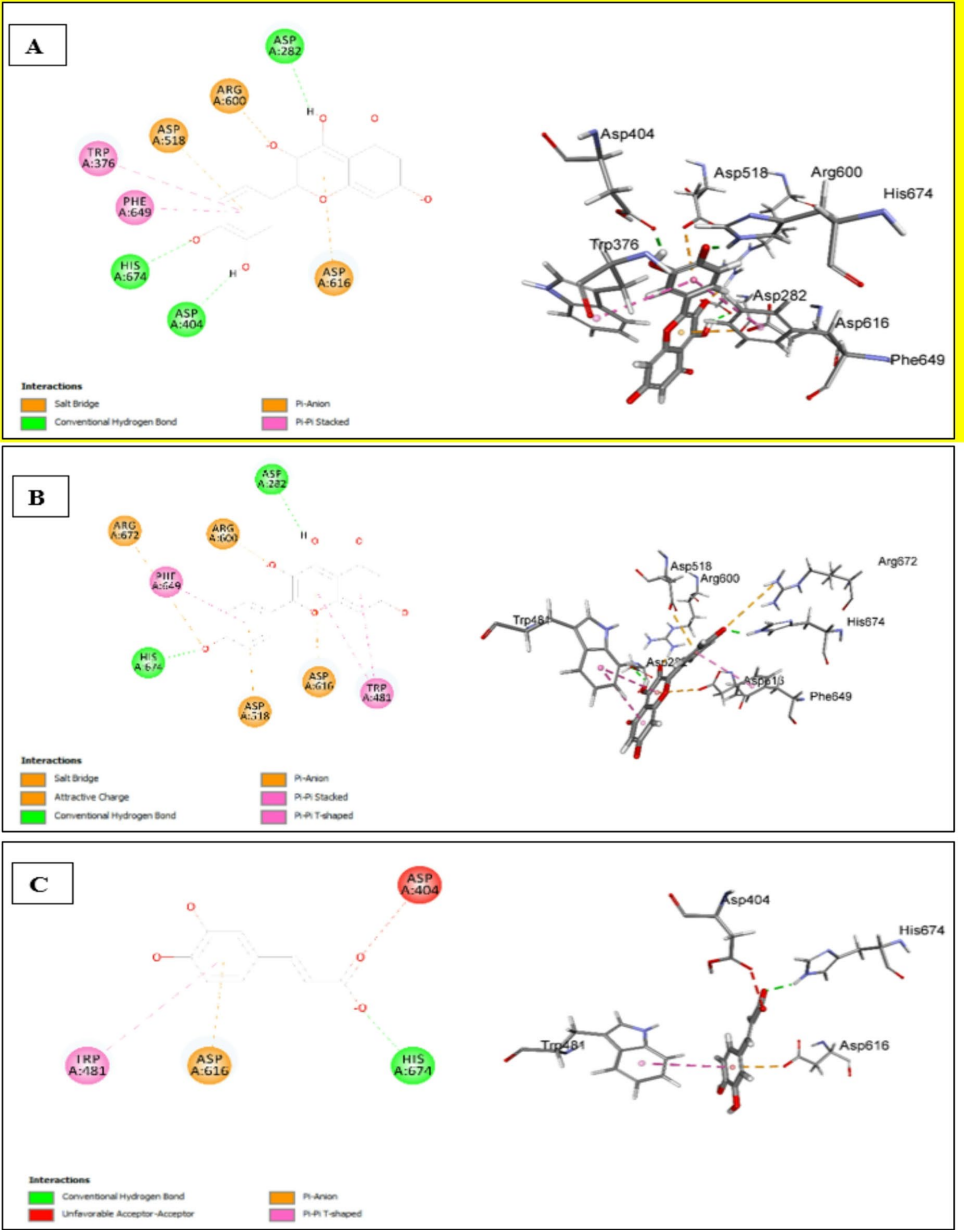
2D & 3D interactions of quercetin (**A**), kaempferol (**B**) and caffeic acid (**C**) with 5NN5 *α*-glucosidase

Quercetin and Kaempferol show almost similar interactions (Fig. 8A, B). They make conventional hydrogen bonds with ASP282 and HIS674, electrostatic interactions with ARG600 and ASP616, and pi-pi interactions with PHE649. While caffeic displayed conventional hydrogen bond and pi-anion interaction, but with different amino acids except for a common conventional hydrogen bond interaction with HIS674. Further, caffeic acid exhibited additional pi-*pi* interaction with TRP481 (Fig. 8C). kaempferol-*O*-hexoside shows very weak binding affinity. As illustrated in Table 8, isorhamnetin-*O*-hexoside, isorhamnetin-*O*-hexosyl-*O*-deoxyhexoside, and oleanic acid failed to dock into the active site of the target protein.

**Table 8:** Docking scores of the major identified compounds with *5NN5 α-glucosidase*

No.	Compound	-CDOCKER Score
1	Quercetin	33.7563
2	Kaempferol	27.4563
3	Caffeic acid	21.2404
4	Oleic acid	18.287
5	kaempferol-*O*-hexoside	− 95.9139
6	Isorhamnetin-*O*-hexoside	Failed to dock
7	Isorhamnetin-*O*-hexosyl-*O*-deoxyhexoside	Failed to dock
8	Oleanic acid	Failed to dock

## Conclusions

The current study assessed the phenolic profile, chemical composition, antioxidant and enzyme inhibitory activities of *C. myxa* leaves and fruits growing in Egypt. The total phenolic and flavonoids content was higher in the leaf extract (71.73 mg GAE/g; 13.85 mg RE/g) than fruit extract (17.51 mg GAE/g; 0.48 mg RE/g). The UPLC-MS^n^ technique was applied to characterize the chemical constituents of both extracts. Different phytochemical classes like phenolic acids, flavonoids, phenolic glycosides, lignans, anthocyanins and fatty acids were identified. The most potent antioxidant activity towards DPPH, ABTS^+^ radicals, reducing power of cupric ions, and chelating of metals were detected in the leaf extract, which was justified by a high predominance of phenolic and flavonoid content. On the other hand, the leaf extract possessed greater BChE (1.68 ± 0.09 mg GALAE/g), amylase (0.16 ± 0.02 mmol ACAE/g), and glucosidase (1.71 ± 0.17 mmol ACAE/g) inhibitory effects than the fruit extract. Meanwhile, the AChE and tyrosinase inhibitory effects were higher in the fruit extract with values of 2.59 ± 0.01 mg GALAE/g and 65.04 ± 1.87 mg KAE/g, respectively. The molecular docking analysis showed that quercetin, kaempferol, and caffeic acid significantly fit to the active sites of targeted enzymes in terms of the highest CDOCKER scores. It can be concluded that the activity against the tested target is attributed to the first six compounds mentioned above, which are quercetin, kaempferol, caffeic acid, oleic acid, kaempferol-*O*-hexoside, and isorhamnetin-*O*-hexosyl-*O*-deoxyhexoside (except 5NN5 α-glucosidase). Accordingly, *C. myxa* leaves signify an excellent natural supply of treasured bioactive constituents with promising antioxidant and enzyme inhibitory effects. Further phytochemical studies should be conducted to isolate the responsible compounds and investigate their mechanism of action, conjugated with pharmacokinetic and pharmacodynamic characteristics.

## Electronic supplementary material

Below is the link to the electronic supplementary material.

Supplementary Material 1

## Data Availability

Data are available upon request from the first author.

## Abbreviations

ABTS: 2,2′-azino-bis(3-ethylbenzothiazoline-6-sulfonic acid
ACAE: Acarbose equivalent
AChE: Acetylcholinesterase
BChE: Butyrylcholinesterase
CMF: *Cordia myxa* fruits
CML: *Cordia myxa* leaves
CUPRAC: Cupric reducing antioxidant capacity
DPPH: 2,2-Diphenyl-1-picrylhydrazyl
EDTAE: Ethylenediaminetetraacetic acid equivalent
FRAP: Ferric reduction antioxidant potential
GAE: Gallic acid equivalent
GALAE: Galanthamine equivalent
MCA: Metal chelating activity
PBD: Phosphomolybdenum
QE: Quercetin equivalent
RDA: Retro-Diels–Alder
RE: Rutin equivalent
ROS: Reactive oxygen species
TE: Trolox equivalent
UPLC-MS^n^: Ultra Performance Liquid Chromatography coupled with tandem mass spectrometry
